# Reliance on shallow soil water in a mixed-hardwood forest in central Pennsylvania

**DOI:** 10.1093/treephys/tpv113

**Published:** 2015-11-06

**Authors:** Katie P. Gaines, Jane W. Stanley, Frederick C. Meinzer, Katherine A. McCulloh, David R. Woodruff, Weile Chen, Thomas S. Adams, Henry Lin, David M. Eissenstat

**Affiliations:** 1 Department of Ecosystem Science and Management, Pennsylvania State University, University Park, PA 16802, USA; 2 Department of Horticulture, Pennsylvania State University, University Park, PA 16802, USA; 3 USDA Forest Service, Pacific Northwest Research Station, Corvallis, OR 97208, USA; 4 Department of Botany, University of Wisconsin, Madison, WI 53706, USA

**Keywords:** Critical Zone Observatory, ecohydrology, ecophysiology, root ecology, rooting depth, sap flux, stable isotopes, tree water use

## Abstract

We investigated depth of water uptake of trees on shale-derived soils in order to assess the importance of roots over a meter deep as a driver of water use in a central Pennsylvania catchment. This information is not only needed to improve basic understanding of water use in these forests but also to improve descriptions of root function at depth in hydrologic process models. The study took place at the Susquehanna Shale Hills Critical Zone Observatory in central Pennsylvania. We asked two main questions: (i) Do trees in a mixed-hardwood, humid temperate forest in a central Pennsylvania catchment rely on deep roots for water during dry portions of the growing season? (ii) What is the role of tree genus, size, soil depth and hillslope position on the depth of water extraction by trees? Based on multiple lines of evidence, including stable isotope natural abundance, sap flux and soil moisture depletion patterns with depth, the majority of water uptake during the dry part of the growing season occurred, on average, at less than ∼60 cm soil depth throughout the catchment. While there were some trends in depth of water uptake related to genus, tree size and soil depth, water uptake was more uniformly shallow than we expected. Our results suggest that these types of forests may rely considerably on water sources that are quite shallow, even in the drier parts of the growing season.

## Introduction

Effective rooting depth, or depth of water uptake, can affect plant productivity and the length of the growing season (Chapin et al. 2002), in addition to playing an important role in determining drought stress (Milly and Dunne 1994, Sternberg et al. 1996, Hubbert et al. 2001), plant competition (Fernandez and Caldwell 1975, Sala et al. 1989, Schwinning 2010), soil formation (Jenny 1980, Schenk 2005) and climate (Kleidon and Heimann 2000, Kleidon and Lorenz 2001). Although the majority of roots are likely to be distributed in the top 30 cm of soil (Jackson et al. 1996, Schenk and Jackson 2002*a*), deep roots have been observed in many water-limited ecosystems and in drought-adapted species (Sternberg et al. 1996, Schenk and Jackson 2002*b*). In some instances, deep roots and roots in fractured bedrock and other barriers have also been observed in forests of the eastern USA (Stringer et al. 1989, Dawson 1993, Canadell et al. 1996). The lack of data from humid temperate forests has limited our understanding of the controls on depth of water extraction in these ecosystems.

Models of root distribution in relation to water uptake suggest that shallow roots predominate in systems with frequent precipitation and abundant shallow soil water (Adiku et al. 1996, Roose and Fowler 2004, Schenk 2005, 2008, Guswa 2008). Accordingly, forests in humid temperate climates may have little need to rely on deep roots if precipitation is frequent. However, the occurrence of random droughts during the growing season even in humid forests (NOAA 2014) and the existence of dry microsites on rocky hillslopes and ridge tops could lead to a fitness advantage for deep-rooted trees (Cavender-Bares and Bazzaz 2000) capable of extracting deep soil water or water held within fractured bedrock (Schwinning 2010). Even small numbers of deep roots could be important for hydraulic redistribution (Dawson 1993, Caldwell et al. 1998) and tree survival (Schenk and Jackson 2002*b*).

Soil depth can shape soil water storage and thus play a role in water availability for transpiration; however, soil depth is unlikely to directly drive root distributions in systems with ample precipitation (Schwinning 2010). Although deep rooting is unlikely to be hindered by shallow soils where fractured bedrock exists, the correlation of topography with soil characteristics could be important for shaping effective rooting depth and for improving estimates of rooting depth for modeling purposes. For example, soil depth could be correlated with water or nutrient conditions that favor or inhibit root growth.

Tree species and their respective genera may be important determinants of rooting patterns and interact with site conditions to determine the depth of water uptake. Among species occurring in central Pennsylvania, *Quercus* (including *Quercus alba* L., *Quercus prinus* L. and *Quercus rubra* L.) tend to be deeply rooted, often with a taproot present early in development that disappears at or before maturity. *Carya* spp., such as *Carya glabra* Mill. and *Carya tomentosa* Nutt., tend to have deep taproots that persist throughout developmental stages. In contrast, individuals from the species *Acer saccharum* Marsh. more heavily depend on lateral roots and are sensitive to flooding. There tends to be more variability in rooting patterns in some genera, such as *Pinus*. For example, *Pinus strobus* L. may have a taproot in favorable soil conditions, while *Pinus virginiana* Mill. tends to be shallow rooted (Burns and Honkala 1990).

Stable isotopes of oxygen (ratio of ^18^O to ^16^O) and hydrogen (ratio of ^2^H to H) in water can be used to integrate the study of plant water fluxes with water in the environment, leading to a better understanding of the role of plants in catchment hydrology. For example, concurrent measurements of the isotopic composition of xylem and soil water can provide estimates of the proportion of water plants use from various water sources as well as the effective rooting depth if the isotopic composition of water sources at different depths is distinct (Dawson and Ehleringer 1998). On similar soils, more enriched xylem water isotopic compositions indicate shallower water sources, whereas less enriched compositions indicate deeper water sources, depending on either an evaporative signal through the soil profile (Barnes and Allison 1983) or seasonal variation in isotopic compositions of precipitation based on temperature. As water from precipitation infiltrates the soil profile, a depth-dependent signal can develop. For example, during the summer growing season, deeper water tends to have a signal similar to winter precipitation, while shallower water tends to reflect the more enriched summer precipitation inputs.

The majority of work using stable isotopes in plants has been conducted in seasonally dry or arid environments (Dawson and Ehleringer 1991, Ehleringer et al. 1991, Flanagan and Ehleringer 1991, Smith et al. 1991, Busch et al. 1992, Valentini et al. 1992, Thorburn and Walker 1993, Mensforth et al. 1994, Meinzer et al. 1999, Cook and O'Grady 2006, Eggemeyer et al. 2009, Brooks et al. 2010, Goldsmith et al. 2012). Until recently (e.g., Brooks et al. 2010, Goldsmith et al. 2012), few studies have combined the study of water use by vegetation using stable isotopes with catchment hydrology (Dawson and Ehleringer 1998).

Current hydrologic models developed at the Susquehanna Shale Hills Critical Zone Observatory (SSHCZO 2015) rely on a fixed rooting depth parameter of 60 cm across all vegetation types and topography in the catchment (Shi et al. 2013). We sought to identify whether this parameter was sufficient to capture the variation in tree rooting depth for water uptake, given the heterogeneous terrain and mixed tree species present at the site. We used measurements of the natural abundance of stable isotopes in soil and xylem water, soil moisture at depth and sap flux to study the patterns of depth of water extraction in trees of different genera and sizes at various slope positions in the Shale Hills catchment in central Pennsylvania. We addressed the following questions: (i) To what extent do trees in our study system rely on deep roots to maintain sap flux during dry portions of the growing season? (ii) What is the role of genus, tree size and slope position or soil depth on depth of water extraction?

## Materials and methods

The primary focus of this study was to understand effective rooting depth of trees for water uptake at the Shale Hills Critical Zone Observatory using natural abundance stable isotopes. To help interpret the isotope data, we used ancillary data from the Critical Zone Observatory. Although we intended to collocate soil water and tree water observations in time and space through the use of lysimeter soil water samples, the existence of multiple, isotopically distinct, soil water pools (see McDonnell 2014) was not something that we originally anticipated. As a result, a caveat of this study was the timing of the bulk soil water samples relative to tree water sampling. Every effort was made, however, to select bulk soil water and additional data from areas with similar topography and soils to the trees sampled in the main study years. As part of our sampling scheme, we used slope position categories based on elevation in order to identify locations in the catchment that were likely to share similar soil conditions. The methods that follow were part of an iterative process that increased our understanding of effective rooting depth in this catchment and the approximate average depth of water use by trees.

### Study site

**Figure 1.**
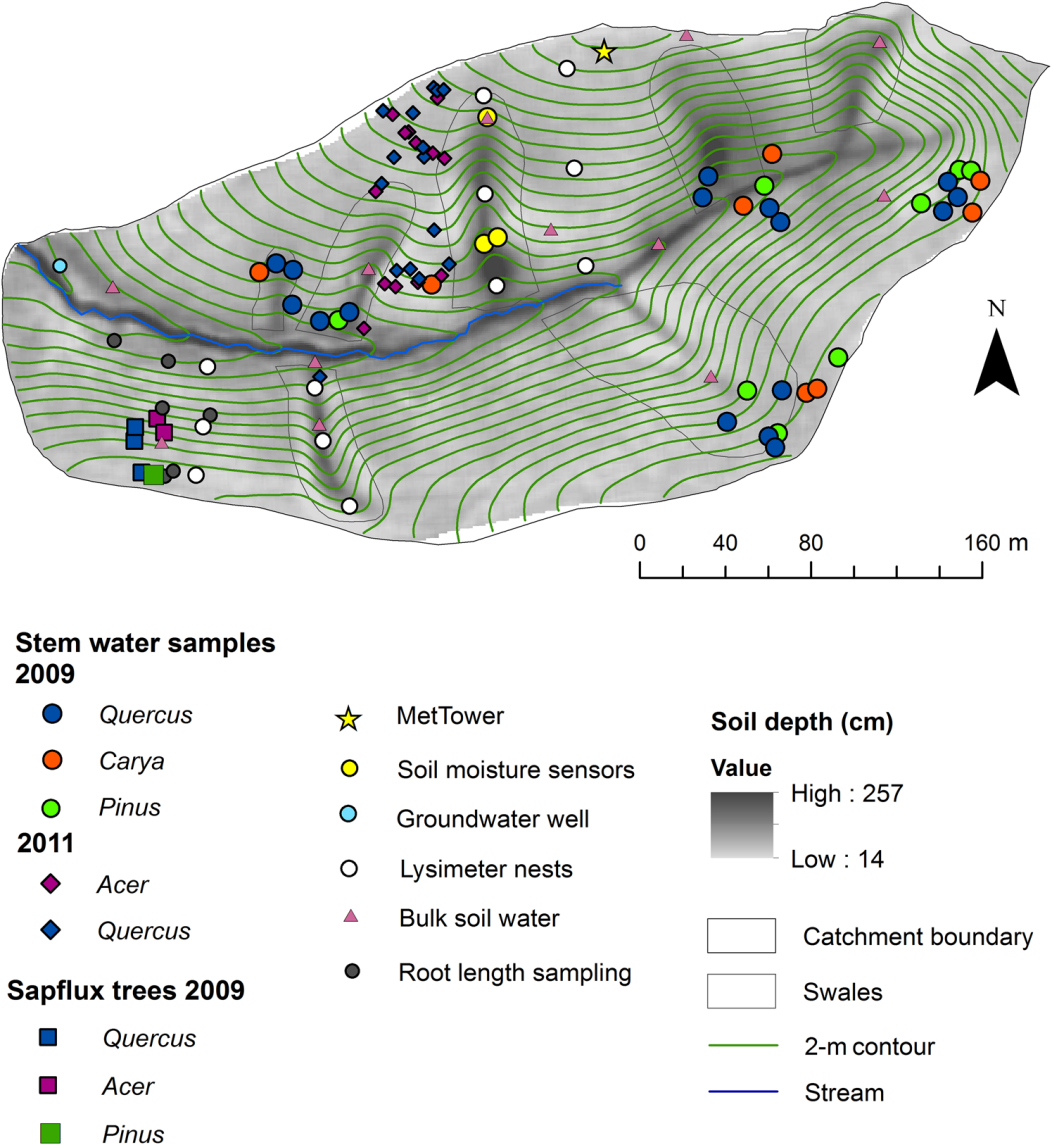
Study area and trees sampled. Points represent locations of isotope sampling including trees sampled for stem water in 2009 and 2011, groundwater (average of 2009 and 2011 groundwater used for calculations), lysimeter nests for mobile soil water sampling (2009 and 2011; 10–340 cm depths), sites of coring for bulk soil water samples (2012) at 10, 20 and 30 cm depth and sites for root length density sampling (2013; 10 cm to up to 140 cm deep). Sensor locations are also shown for soil moisture (volumetric water content from 2009; up to 162 cm deep) and sap flow (2009; south ridge site). Precipitation isotopes and amount (2009 and 2011) were measured using equipment near the meteorological tower on the north ridge top.

The Shale Hills Critical Zone Observatory (Shale Hills; Lat. 40°39′ N, Long. 77°54′ W, elevation 256–310 m) was chosen as a study site because of the well-characterized topography, soils and vegetation and the distribution of different individuals of the same genera in areas with contrasting soil depth (Figure 1) and other soil conditions. Shale Hills is a 7.9-ha catchment within Penn State University's Stone Valley Research Forest. The catchment is v-shaped with north- and south-facing slopes, and a valley floor serving as a floodplain for the stream that runs east to west. The forest was last harvested for timber in the 1930s and has been used for research purposes since the 1970s (Naithani et al. 2013). In 2008, the forest was surveyed for trees over 18 cm in diameter at breast height (DBH), species and crown class. Additional diameter and height measurements were taken in subsequent years to record growth, and the survey was updated in 2012. Forest composition on a basal area basis predominantly consisted of the hardwoods *Quercus*, *Carya* and *Acer*, in addition to conifers of *Tsuga* and *Pinus*.

Average annual precipitation for the site was 1006 mm, which is evenly distributed throughout the year (Thomas et al. 2013). Despite the frequent precipitation that is typical in this region of Pennsylvania, hydrologic droughts do occur occasionally during the growing season. Moderate to extreme drought has occurred at some point from the months of May through August during ∼29% of the last 114 years, with most of these events lasting less than a month (NOAA 2014).

### Soils

Soils at the site were derived from shale colluvium or residuum with many shale fragments throughout soil profiles, particularly on hillslopes and ridge tops. Ridge and hillslope soils were well-drained silt loam, while the valley bottom had fine-loamy soils. Due to seasonally saturated conditions, valley soils had redoximorphic features at ∼0.3–0.5 m depth (Lin et al. 2006, Thomas et al. 2013). Soils on the valley floor tended to have higher clay content and lower sand content in the soil profile compared with other slope positions (Baldwin 2011). Soil depth was measured by augering at 223 points throughout the catchment and represents the soil depth to refusal (Lin et al. 2006). A layer of fractured bedrock below the soil can be many meters deeper than the depth of refusal (Jin et al. 2011*a*). Soil depth for each individual tree was estimated by extracting values of soil depth at tree locations from a map layer in GIS software (ArcGIS 10.0; ESRI 2010).

### Root length density

Cores were collected at three slope positions (ridge, midslope and valley) in August 2013 to assess differences in root length density (length of root per ground surface area; cm cm^−2^) with slope position. Within these locations, two nests of cores were sampled with three replicates each. Cores were collected at depth increments of 0–10, 10–20, 20–40 and 40+ cm. The last depth increment was based on depth to refusal of the coring equipment. On some valley floor locations, an ending depth of 100 cm was selected based on equipment limitations in the heavy clay soil. A manual soil tube and associated equipment were used (ST-140, Giddings Machine Company, Windsor, CO, USA). The soil cores were kept refrigerated for up to 1 month until the roots could be washed using a 2-mm mesh sieve.

First- and second-order fine roots were separated from higher order roots and scanned using a desktop scanner with WinRHIZO software (WinRHIZO Pro, Regent Instruments, Inc., Quebec, QC, Canada) for length measurements. The depth increment of each sample and inner core radius were used to calculate soil volume for each sample to compare root lengths at different soil depths.

### Isotopic composition of water sources

The potential water sources for trees in the Shale Hills catchment (precipitation, soil water and groundwater) were examined and compared with tree xylem water δ^2^H and δ^18^O compositions, to infer both water sources and depth of water uptake by trees.

### Precipitation

Precipitation was measured at the meteorological tower site on the north ridge (Figure 1) with an Ott-Pluvio weighing bucket (Hach Company, Loveland, CO, USA) with additional precipitation measurements from a Laser Precipitation Monitor (LPM, Thies Clima, Gottingen, Germany) and tipping-bucket rain gauges (Davis Instruments Corp., Hayward, CA, USA) used to fill in missing data (Thomas et al. 2013). Precipitation was collected using an automatic precipitation sampler (NSA 181/S model, Eigenbrodt GmbH & Co., Konigsmoor, Germany) in a clearing on the north ridge (Figure 1) on an event basis, using the methods described in Thomas et al. (2013). Isotopic compositions for 2009 and 2011 were integrated by the amount of each rain event over the course of a season and averaged together to determine seasonal average δ^18^O and δ^2^H compositions. The weighted local meteoric water line (LMWL) for the years 2008–11 from the Shale Hills catchment (Thomas et al. 2013) was examined in relation to the tree water and source water compositions.

### Mobile soil water

Mobile soil water was sampled at 12 lysimeter nests within the Shale Hills catchment (Figure 1) during the growing season in 2009 and 2011 (Jin et al. 2011*b*, Thomas et al. 2013). Two transects with lysimeter nests at ridge top, midslope and valley floor locations were located on the north slope and two on the south slope. Within those transects, one was located on a planar hillslope (convex curvature) and the other on a swale (concave curvature). Lysimeters were sampled between 10 and 340 cm, depending on the depth to refusal at the location of the nest. Depths ≤100 cm were sampled every 10 cm, and depths >100 cm were sampled at intervals between 10 and 40 cm. Samples were collected typically every 1–3 weeks. Soil water was collected from lysimeters using a hand pump to sample water held at tensions up to ∼50 kPa. As a result, during dry parts of the year (June–September), lysimeters were not able to be sampled as frequently due to low soil matric potentials. Additional details on lysimeter locations and depths are found in Thomas et al. (2013).

### Bulk soil water and groundwater

Due to the limited availability of lysimeter samples during the driest part of the year (late summer), and preliminary results suggesting that the mobile water was not the most likely water source for trees at this time, soil coring and sampling for more tightly bound ‘bulk’ soil water were also conducted at a later date. Twelve soil cores were collected from 12 sites from 5 swale and 7 planar hillslope locations in July 2012: with 3 sites on the valley floor, 5 on midslope position and 4 on the ridge (Figure 1). Soil cores were collected manually using a hand auger (AMS, Inc., American Falls, ID, USA) within 3 m of soil moisture monitoring stations at 10 cm (0–10 cm), 20 cm (10–20 cm) and 30 cm (20–30 cm) depths. A glass vial was inserted into the bottom of the auger to extract soil with minimal exposure to air. Samples were sealed with a Polyseal cap, wrapped with Parafilm and kept in an insulated container before being returned to the laboratory for storage at 0 °C until water was extracted and analyzed for isotopes (see below).

A groundwater water well in the riparian zone at Shale Hills was sampled daily (2700 series, Teledyne Isco, Lincoln, NE, USA; Figure 1) at a depth of ∼2.74 m (Duffy and Thomas 2011*a*).

### Tree sampling

Tree xylem water samples were collected in 2009 and 2011 from a total of 60 trees (Table 1, Figure 1) representing the most dominant genera in the catchment that were also widely distributed throughout forests of the eastern USA. Trees from the species *C. glabra*, *C. tomentosa*, *P. strobus*, *P. virginiana*, *Q. alba*, *Q. rubra*, *Q. prinus* and *Q. velutina* were sampled in 2009 from sites with contrasting slope positions and soil moisture levels. *Acer saccharum* and *Q. prinus* were sampled in 2011 along a transect from ridge to valley floor on the south-facing slope. Trees were sampled between one and four times over the course of the growing season (May–October). Each tree was measured for DBH and height (TruPulse 360, Laser Technology, Inc., Centennial, CO, USA).

**Table 1.** Summary of tree characteristics. Tree genus, species, number of individuals sampled, DBH (cm), tree height (m) and average estimated soil depth at location of trees sampled. Standard error of the mean values is given in parentheses.

Genus	Species	*n*	DBH (cm)	Height (m)	Soil depth (cm)
*Acer*	*A. saccharum*	13	23.0 (2.5)	16.2 (0.8)	44 (6)
*Carya*	*C. glabra*	4	29.4 (3.5)	20.4 (1.5)	46 (7)
	*C. tomentosa*	4	33.8 (5.0)	23.6 (2.4)	64 (15)
*Pinus*	*P. strobus*	4	33.4 (1.8)	24.3 (2.7)	66 (24)
	*P. virginiana*	4	32.9 (3.5)	18.5 (0.2)	28 (7)
*Quercus*	*Q. alba*	4	34.5 (3.0)	24.6 (3.2)	56 (14)
	*Q. prinus*	21	36.8 (1.9)	20.3 (0.7)	44 (5)
	*Q. rubra*	4	46.3 (5.2)	26.7 (2.0)	56 (11)
	*Q. velutina*	2	49.8 (2.8)	28.9 (0.3)	119 (15)

Samples for stable isotopes were obtained from canopy height branches of ∼50 cm length. Small segments of fully suberized, woody branch samples of ∼3–5 mm in diameter were cut and quickly sealed in glass vials with Polyseal caps and Parafilm to prevent water loss. Samples were kept shaded and transported back to the laboratory the same day (within 3 h) and frozen for long-term storage.

### Soil moisture depletion

Three sites on a hillslope swale were chosen for volumetric water content measurements (ECH2O 10 cm probes and 5TE probes, Decagon Devices, Inc., Pullman, WA, USA) to estimate the soil water conditions over a deep soil profile (>1 m) during dry periods in 2009 (Figure 1). Between 12 and 16 sensors were installed at each of these three sites between 5 and 162 cm depth. These sites on a hillslope swale were chosen because data below 40 cm depth were not available for planar hillslope or ridge locations as shale fragments and fractured regolith prevented deeper sensor installation. Water contents were estimated using the midpoint between sensor depths as a weighting factor, then totaled to represent the amount of water over the soil profile. Shallow soil water (<40 cm deep) and deep soil water (>40 cm deep) were compared to determine relative contributions of water in these soil layers to tree sap flux. In addition, relative volumetric water contents were analyzed for the same three hillslope soil moisture sites and blocked according to depth increment during periods without rain. For each period without rain lasting at least 5 days (‘dry cycle’) and subsequent recovery, the maximum daily volumetric water content was calculated as a percentage of the maximum water content during the study period.

### Sap flux

Fixed and variable depth heat dissipation sap flow probes were installed in trees of the dominant genera at Shale Hills in 2009 and 2010 (see Meinzer et al. 2013). A regression technique was used to estimate the temperature difference between probes at the point of zero flow (Δ*T*_max_) (Meinzer et al. 2013). To characterize general patterns in sap flux in relation to soil moisture for a dry area of the catchment, observations from multiple sensors on individual trees were averaged together. Observations of sap flux when vapor pressure deficit (VPD) was very low (<0.1 kPa) were excluded from the analysis so as to avoid bias in our calculation of mean daily sap flux (Phillips and Oren 1998). Mean daily values (between 7 am and 7 pm) were normalized by the maximum for each tree over the week of the dry cycle. Sap flux was examined for one dry cycle in 2009 for trees in the genera *Acer* (*n* = 2), *Quercus* (*n* = 3) and *Pinus* (*n* = 1) on a ridge site (Figure 1). This dry cycle was just one of three dry cycles for that year; sap flux data were not available for earlier in the season. Sap flux data for six individuals (*Quercus*, *n* = 4; *Acer*, *n* = 2) on a ridge top site over three dry cycles during 2010 were also analyzed. To estimate mean daily crown conductance for individuals sampled in 2009, mean daily sap flux for individual trees was divided by mean daily daytime VPD with appropriate unit conversions. Mean daily sap flux for the beginning of the dry period (day of year (DOY) 234–235) was compared with the end of the dry period (DOY 237–238) in order to examine whether there were changes in sap flux as a result of soil moisture depletion.

### Analytical approaches

Water from tree and bulk soil samples was extracted by cryogenic vacuum distillation prior to analysis (West et al. 2006). Tree water and bulk soil water samples that underwent cryodistillation were analyzed at the Center for Stable Isotope Biogeochemistry (CSIB) at the University of California at Berkeley using continuous flow with a gas chromatography system for δ^18^O (GasBench II, ThermoFinnigan, Bremen, Germany) and by dual inlet using a hot chromium reactor unit for δ^2^H (H/Device, ThermoFinnigan). Both analytical techniques were coupled to an isotope ratio mass spectrometer (Thermo Delta Plus XL, ThermoFinnigan). The long-term external precision of these methods was ±0.80‰ for δ^2^H and ±0.12‰ for δ^18^O (standard deviation) (CSIB 2014).

Source water samples collected without cryodistillation (precipitation, groundwater and mobile soil water) were analyzed for δ^18^O and δ^2^H by a LGR DLT-100 Liquid Water Isotope Analyzer (Los Gatos Research, Inc., Mountain View, CA, USA) using International Atomic Energy Agency standard operating procedures (standard deviation of ±0.3‰ for δ^18^O and ±0.8‰ for δ^2^H) (Lis et al. 2008). The ratios of ^18^O to ^16^O and ^2^H to H were calculated relative to Vienna Standard Mean Ocean Water and expressed in delta (δ) notation in parts per thousand (‰):

(1) $$X\text{‰}=\left(\left[\left(\frac{R_{\mathrm{sample}}}{R_{\mathrm{standard}}}\right)\right]-1\right)$$

where *R* is the ratio of heavy to light isotope (^18^O/^16^O or ^2^H/H).

### Effective rooting depth model

Trees were assigned to slope position categories, based on similarities in elevation to lysimeter nests at valley floor (266–268 m), midslope (269–291 m) and ridge (292–300 m) positions. δ^18^O and δ^2^H signatures were separately plotted against soil depth for bulk soil water samples at these three slope positions (see Figure S1 available as Supplementary Data at *Tree Physiology* Online). The *y*-intercepts of the linear regression lines were assumed to represent the soil water signature at a depth of 0 cm. Therefore, this isotopic signature at 0 cm depth was used as one end member for each individual slope position category.

Depth of water extraction by trees was estimated using δ^18^O and δ^2^H with a linear mixing model (Thorburn and Walker 1993).

(2) $$D_{s-g}=[(X_{s}-X_{g}{)}^{2}+(Y_{s}-Y_{g}{)}^{2}]$$

(3) $$D_{s-t}=[(X_{s}-X_{t}{)}^{2}+(Y_{s}-Y_{t}{)}^{2}]$$

(4) $$P_{g}=\frac{D_{s-t}}{D_{s-g}}$$

where *D*_s–g_ is the isotopic distance or difference between isotopic compositions between surface soil water (s) and groundwater (g) for δ^18^O (*X*) and δ^2^H (*Y*), *D*_s–t_ is isotopic distance or difference between isotopic compositions between surface soil water and tree water (t) and *P*_g_ is the proportion of groundwater in tree water. Depth of water extraction (DW) was then calculated as follows:

(5) $$\mathrm{DW}=P_{g}\times 120$$

Deep soil water (≥120 cm) had less seasonal variation and approached the groundwater isotopic composition during the years of this study (Thomas et al. 2013). As a result, a maximum depth of 120 cm was chosen to represent the depth of groundwater. The average groundwater signature for the study period was used as the second end member in the mixing model.

A second approach was also used in order to examine how rooting depth estimates would change if modeled rooting depth was restricted to the depth of soil water analyzed for this study (30 cm). In this case, 30 cm was used as an end member for this additional analysis.

### Statistical analyses

In order to investigate the main research questions of this study, linear mixed-effects models with δ^18^O and δ^2^H as response variables in separate models, with candidate predictors of tree genus, tree size (DBH and height) and location-specific variables including slope position, soil depth and elevation were used. Tree sample dates with <0.2 mm of rainfall in the preceding 2 days were selected (‘dry dates’). Individual tree was used as a random effect in each candidate model to account for the multiple observations for some of the individual trees in the study. Fixed effects were added one at a time to a random-effect-only model in order of their coefficient of determination. Nested models were compared using Akaike's information criterion (AIC) corrected for small sample size (AIC_c_) and Akaike weights (*w_i_*). The correlations between fitted and observed δ^18^O and δ^2^H results were used to obtain an estimate of the percent of total variation explained by the model to obtain an approximate *R*^2^ value (Byrnes and Stachowicz 2009).

As part of the model building process, univariate models were explored to examine variation in δ^18^O and δ^2^H with each candidate variable, as well as correlations among candidate variables. One-way analysis of variance (ANOVA) was used to explore the data for systematic differences between tree genera with respect to soil depth and tree size (DBH and height), and to examine differences in soil depth between slope positions.

As a secondary measure to understand patterns in tree xylem δ^2^H and δ^18^O, a paired *t*-test was used to assess genus-level differences in δ^2^H and δ^18^O independent of location within the catchment by matching up trees of different genera that were sampled on the same dates in close spatial proximity to one another.

Univariate models were explored with effective rooting depth and the variables outlined above. A 95% confidence interval was calculated for the grand mean effective rooting depth estimate. A mixed effect model was not used to examine controls on effective rooting depth due to the derivation of the model from slope position-specific parameters, and instead variation of effective rooting depth and tree genus was evaluated with ANOVA.

One-way ANOVA was used to test for differences in root length density among slope positions, and linear regression models were used to test for the effect of time since rainfall on volumetric water content. Statistical analyses were conducted using R base packages and the lme4 package in R (R Development Core Team 2015). Averages reported represent mean ± standard error unless otherwise noted.

## Results

### Environmental conditions

**Figure 2.**
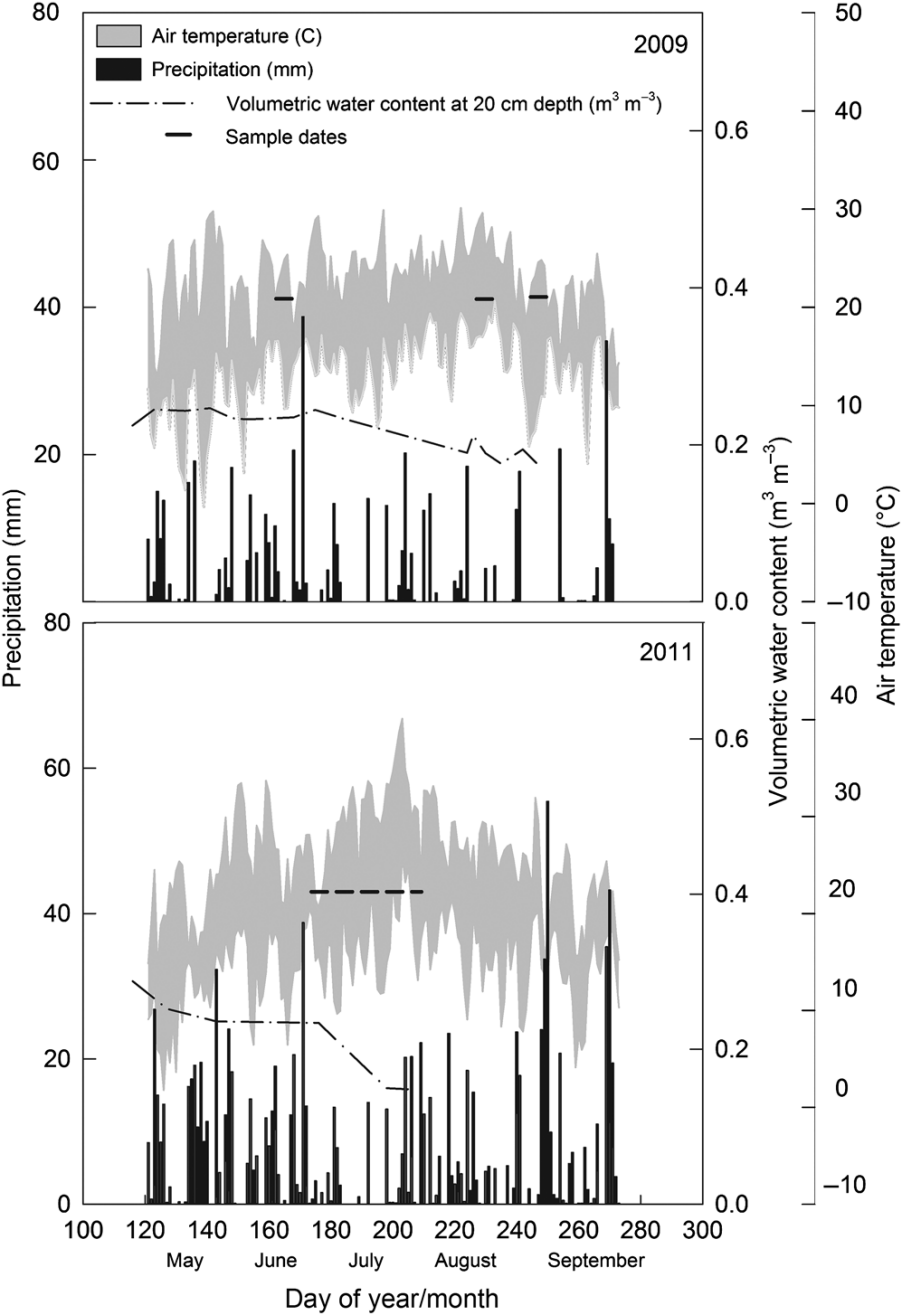
Environmental conditions during 2009 and 2011. Precipitation amount (mm) in black bars on primary *y*-axis (left), volumetric water content at 20 cm depth (m^3^ m^−3^) on secondary axis (right) and air temperature (°C) minimum and maximum daily values on tertiary *y*-axis (far right). Sample dates for data shown in remainder of results are displayed with dashed horizontal lines.

Average air temperatures for this region of Pennsylvania from May to September are ∼18 °C with precipitation of ∼94 mm per month (NOAA 2015). Weather conditions were in the average range during the growing season of 2009, but were wetter overall in 2011 (NOAA 2014, Figure 2). Annual precipitation in 2009 was 1033 and 1321 mm in 2011. While there was no precipitation in July of 2011, leading to sharp declines in soil moisture during that month, the beginning and end of the growing season were quite wet. Rainfall in 2009 occurred regularly throughout the summer with average soil moisture decline beginning in early July (DOY = 182). Sample dates, indicated by the solid horizontal lines in Figure 2, were chosen from late June to early September in order to represent periods likely to coincide with soil moisture deficits as reflected in reduced volumetric water content (dash-dot line in Figure 2). In contrast, central Pennsylvania experienced moderate hydrologic drought from June to September of 2010 (NOAA 2014).

### Tree and site characteristics

**Figure 3.**
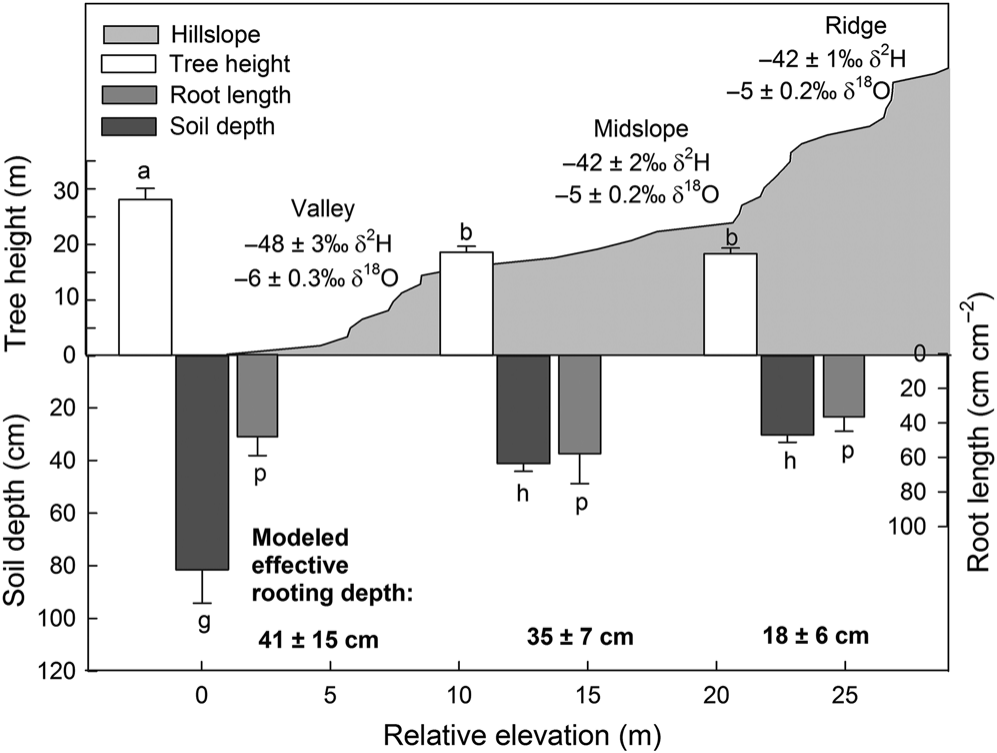
Depiction of Shale Hills catchment and related plant and soil traits with relative slope position and relative elevation. Values shown indicate mean ± standard error. Tree water isotope data shown are for dry dates. Tree height and soil depth at tree locations are for all trees sampled during the study. Tree height (*P* < 0.001) and soil depth (*P* < 0.0001) varied by slope position (differences denoted with letters), while root length did not (*P* = 0.5). Average δ^2^H and δ^18^O compositions shown for trees at each slope position (top), along with effective rooting depth estimates (bottom, shown for illustrative purposes only). Isotopic compositions varied by soil depth, but not by slope position. Error bars represent mean ± standard error.

Average tree size varied among genera, with *Acer* tending to be smaller than some of the other genera (Table 1). Mean DBH and height differed significantly by tree genus, with *Acer* smaller on average than *Quercus* (*P* < 0.01 for DBH and *P* < 0.001 for height). Average height varied by slope position, with the tallest trees on the valley floor and the shortest trees on the ridge and midslopes (*P* < 0.05, Figure 3). Soil depth varied by slope position (*P* < 0.01), with valley soils 41 cm deeper on average than soils on the midslope and 52 cm deeper than soils on the ridge (Figure 3). Accordingly, there was a negative correlation of soil depth and elevation (*r* = 0.40), with trees at higher elevations tending to be located on shallower soil than trees at lower elevations.

### Root length density

**Figure 4.**
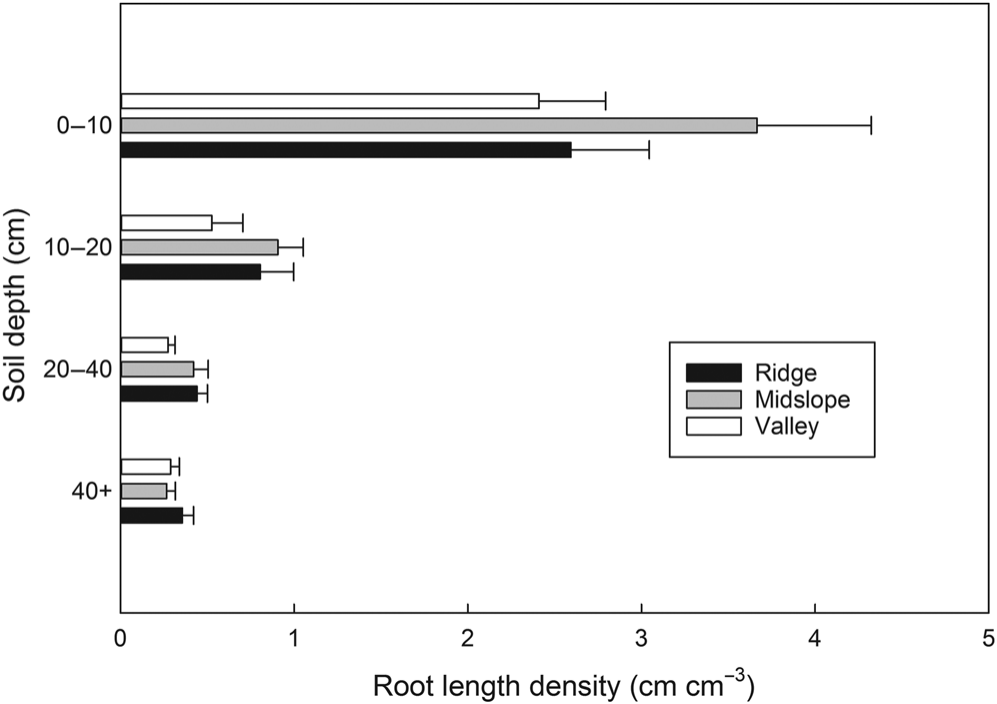
Root length density by depth (per unit volume of soil) for ridge, midslope and valley slope positions (cm cm^−3^). Eighteen cores were collected, six at each slope position, on sites with planar curvature (Figure 1). Error bars represent standard error of the mean. Coring depth for ‘40+’ category was 59 cm (±4 cm) for the ridge sites, 69 cm (±0.8 cm) for the midslope and 62 cm (±2 cm) for the valley sites.

Root length density by depth increment on a volume basis (cm cm^−3^) was highest in the top 10 cm of soil and declined steeply with depth (Figure 4). Total root length per ground surface area did not differ significantly by slope position category (midslope, 56 ± 17 cm cm^−2^; ridge, 48 ± 11 cm cm^−2^; valley floor, 35 ± 8 cm cm^−2^; *P* = 0.52). On average across slope positions, >84% of total root length to depth of refusal was located in the top 40 cm of soil, with over 50% in the top 10 cm.

### Water sources

**Figure 5.**
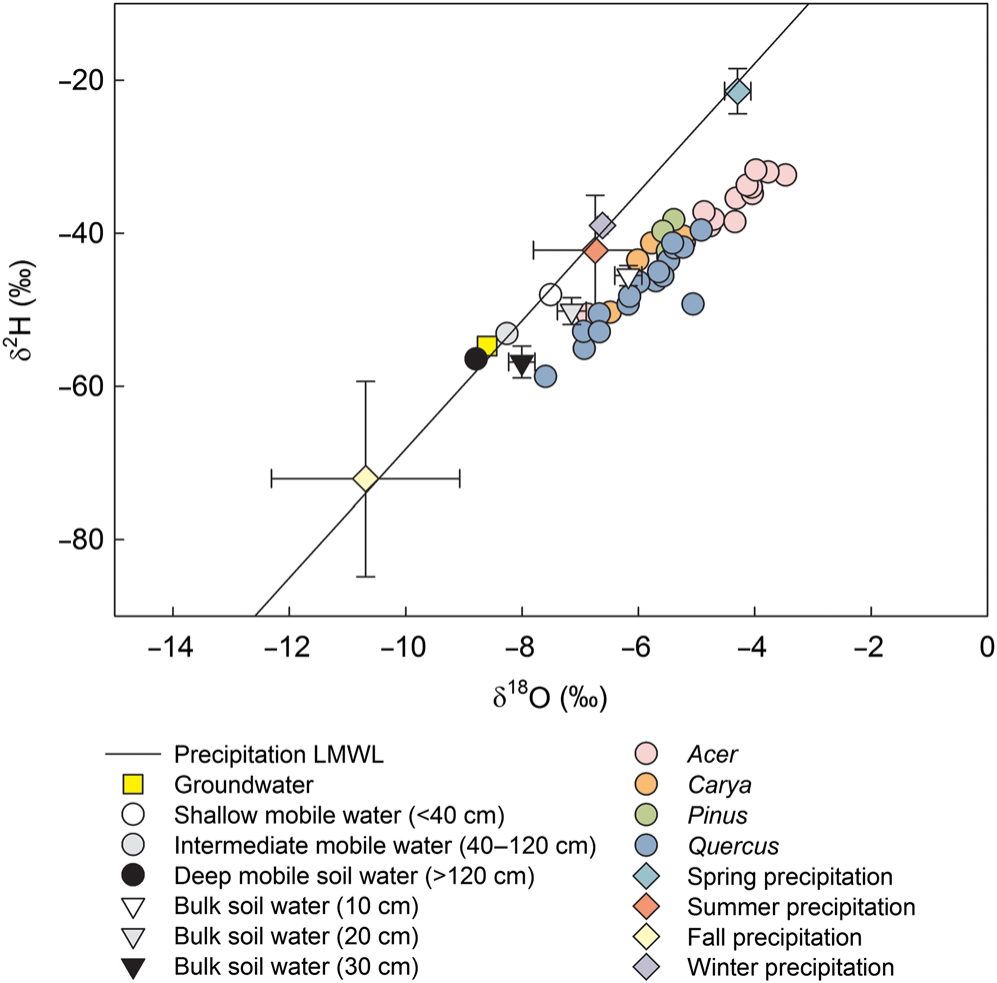
Tree xylem water and potential source water δ^2^H and δ^18^O compositions with an amount-weighted LMWL, including average groundwater, average mobile soil water, average bulk soil water by depth and amount-weighted seasonal precipitation. Error bars represent mean ± standard error. Equation of weighted LMWL: *y* = 8.4*x* + 15.8.

Precipitation δ^18^O and δ^2^H signatures showed a large amount of variation and strong seasonal patterns, with compositions more enriched in heavy isotopes in spring and summer than in fall and winter (Figure 5). The average isotopic composition for the fall season was strongly affected by a large snowstorm in October 2011 that had isotopic compositions very depleted in heavy isotopes. This resulted in an average seasonal value for fall that was more typical of winter precipitation.

During 2009 and 2011, shallow mobile soil water (<40 cm) had an average composition of −48.1 ± 0.7‰ for δ^2^H and −7.5 ± 0.1‰ for δ^18^O, while deep mobile soil water (≥120 cm) had a composition of −56.4 ± 0.4‰ for δ^2^H and −8.8 ± 0.06‰ for δ^18^O (Figure 5). Groundwater had a similar signature to deep mobile soil water with very little seasonal variation. The average groundwater compositions during the study years were −54.6 ± 0.082‰ for δ^2^H and −8.6 ± 0.021‰ for δ^18^O (Figure 5). Soil water sampled from lysimeters (‘mobile’ water) at this study site showed higher variability in isotopic signature in the shallow soil than in the deeper soil on average (Thomas et al. 2013). Standard deviations decreased with depth (12.6‰ for shallow mobile soil water δ^18^O, 6.2‰ for deep soil water δ^18^O and 1.9‰ for groundwater δ^18^O), reflecting a dampening of the seasonal precipitation signature (Thomas et al. 2013). Soil water sampled with lysimeters and groundwater fell on or above the weighted LMWL (Figure 5).

Water distilled from soil cores (bulk soil water) was most enriched in heavy isotopes in the shallow soil (10 cm) (δ^2^H =<−45.6 ± 1.3‰; δ^18^O = −6.2 ± 0.2‰) and least enriched for the deepest layer (30 cm) (δ^2^H = −56.9 ± 2.1‰; δ^18^O = <−8.0 ± 0.2‰) (Figure 5). Bulk soil water fell below the LMWL, showing evidence of evaporative enrichment (Barnes and Allison 1983). The ordering of bulk soil water signals relative to depth was predictable based on slope position categories, with soils on the ridge less depleted in heavy isotopes relative to midslope and valley locations (see Figures S1 and S2 available as Supplementary Data at *Tree Physiology* Online).

### Tree water isotopes

Tree xylem water isotope compositions for dry dates ranged from −7.6 to −3.5‰ for δ^18^O and −58.7 to −31.8‰ for δ^2^H, with a mean of −5.4 ± 0.2‰ for δ^18^O and −42.7 ± 1.1‰ for δ^2^H. Tree water had isotopic compositions that fell below the LMWL (Figure 5). Variation in tree water isotopic composition had a slope that was less steep than the LMWL (slope of 6.5, compared with the weighted LMWL slope of 8.4), with a lower intercept (tree water intercept of −7.6, LMWL intercept of 15.8).

**Figure 6.**
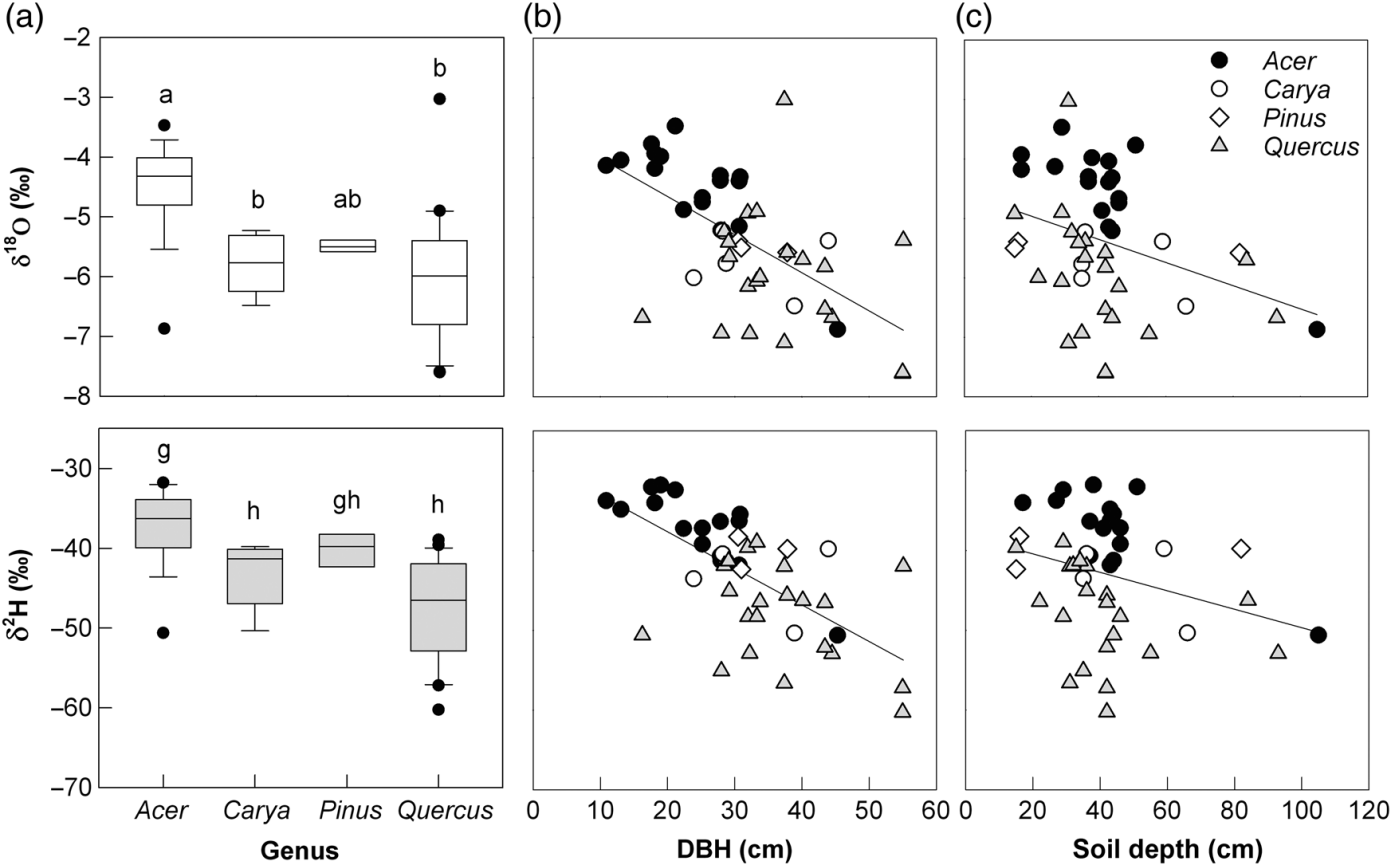
(a) Boxplot of xylem water δ^18^O (top) and δ^2^H (bottom) compositions for genera sampled during dry sample dates. Upper and lower boundaries of box denote 75th and 25th percentile ranges, respectively, with median at center line. Error bars show maximum and minimum range of data with points for observations exceeding 1.5 times the interquartile range. Letters denote statistically significant differences (genus differences in δ^18^O, *P* < 0.0001; δ^2^H, *P* < 0.00001; *Acer*, *n* = 13; *Carya*, *n* = 5; *Pinus*, *n* = 3; *Quercus*, *n* = 17). (b) Scatter plot of tree DBH and tree water δ^18^O (top) and δ^2^H (bottom) compositions for individuals sampled on dry dates (δ^18^O, *P* < 0.00001, *R*^2^ = 0.35, *y* = −0.063*x* − 3.37; δ^2^H, *P* < 0.000001, *R*^2^ = 0.42, *y* = −0.46*x* − 28.45). (c) Scatter plot of soil depth at tree locations and tree water δ^18^O (top) and δ^2^H (bottom) compositions for individuals sampled on dry dates (δ^18^O, *P* < 0.05, *R*^2^ = 0.09, *y* = −0.020*x* − 4.57; δ^2^H, *P* < 0.05, *R*^2^ = 0.07, *y* = −0.12*x* − 38.1).

Univariate tests used in preparation for mixed effect model building showed that genus, tree size and soil depth were all significant predictors of both δ^18^O and δ^2^H (Figure 6). *Quercus* and *Carya* xylem water tended to be more depleted in heavy isotopes than that of *Acer* (overall genus effect, *P* < 0.0001 and *R*^2^ = 0.36 for δ^18^O; *P* < 0.00001 and *R*^2^ = 0.46 for δ^2^H) (Figure 6a). Tree size was a significant negative linear predictor of δ^18^O and δ^2^H, with larger trees tending to use water more depleted in heavy isotopes than smaller trees. Diameter at breast height was used in the model building process since DBH and tree height were positively correlated (*r* = 0.59), and DBH was a stronger predictor of xylem water δ^18^O than tree height (DBH, *P* < 0.00001, *R*^2^ = 0.35; tree height, *P* < 0.001, *R*^2^ = 0.22) and δ^2^H (DBH, *P* < 0.000001, *R*^2^ = 0.42; tree height, *P* < 0.01, *R*^2^ = 0.19) (Figure 6b). Soil depth was a significant linear predictor of δ^18^O (*P* < 0.05, *R*^2^ = 0.09) and δ^2^H (*P* < 0.05, *R*^2^ = 0.07) (Figure 6c), but with lower explanatory power compared with genus and DBH. Slope position and elevation were not significant predictors of δ^18^O or δ^2^H (δ^18^O, *P* = 0.19 for slope position and *P* = 0.91 for elevation; δ^2^H, *P* = 0.36 for slope position and *P* = 0.69 for elevation). A comparison of nested models with a random effect of individual tree and candidate fixed effects (Table 2) resulted in the selection of a final model with all possible fixed effects that we examined (genus, DBH and soil depth; Table 3). The final model explained ∼54% of the variation in tree xylem water δ^18^O and ∼63% of the variation in δ^2^H.

**Table 2.** Linear mixed-effects models evaluated. δ^18^O and δ^2^H were examined separately. Model name in order of least to most complex, equation of model, small sample size AIC_c_ value, model weight (*w_i_*) and degrees of freedom (df). Model d was chosen as the final model for both δ^18^O and δ^2^H.

Model	Equation	AIC_c_	*w_i_*	df
δ^18^O				
a	δ^18^O ∼1 + (1|treeID)	143	4.3 × 10^−7^	NA
b	δ^18^O ∼1 + genus + (1|treeID)	124	6.6 × 10^−3^	3
c	δ^18^O ∼1 + genus + DBH + (1|treeID)	118	0.15	4
d	δ^18^O ∼1 + genus + DBH + soil depth + (1|treeID)	114	0.84	5
δ^2^H				
a	δ^2^H ∼1 + (1|treeID)	309	9.5 × 10^−8^	NA
b	δ^2^H ∼1 + genus + (1|treeID)	289	2.1 × 10^−3^	3
c	δ^2^H ∼1 + genus + DBH + (1|treeID)	280	0.23	4
d	δ^2^H ∼1 + genus + DBH + soil depth + (1|treeID)	278	0.77	5

**Table 3.** Summary of final model fixed effects (Model d) for δ^18^O and δ^2^H. *Carya*, *Pinus* and *Quercus* were dummy variables for genus (reference is *Acer*).

Model	Fixed effect	Estimate	Standard error	*t*-Value
δ^18^O	(Intercept)	−3.3	0.37	−8.88
	Genus			
	*Carya*	−1.0	0.39	−2.60
	*Pinus*	−0.85	0.48	−1.76
	*Quercus*	−1.2	0.30	−3.98
	DBH	−0.02	0.01	−1.61
	Soil depth	−0.02	0.007	−2.25
δ^2^H	(Intercept)	−29.0	2.2	−13.18
	Genus			
	*Carya*	−4.05	2.3	−1.76
	*Pinus*	−1.67	2.8	−0.59
	*Quercus*	−8.60	1.8	−4.80
	DBH	−0.20	0.085	−2.325
	Soil depth	−0.08	0.039	−1.990

An analysis of nine collocated *Acer* and *Quercus* individuals (12 paired observations) sampled on the same days without recent precipitation in 2011 (*P* < 0.0001 for δ^2^H; *P* < 0.01 for δ^18^O) ruled out the possibility that the relative differences in δ^2^H and δ^18^O composition were simply due to variation in soil isotopic composition from different locations within the catchment. Compared with *Acer* individuals in this analysis, *Quercus* individuals had 12.65‰ more depleted δ^2^H and 1.62‰ more depleted δ^18^O compositions.

### Modeled effective rooting depth

The grand mean effective rooting depth was 32 cm with a 95% confidence interval of 15–61 cm, providing a rough estimate of the likely depth of water uptake by the study trees during the growing season. For both years combined, we were unable to detect significant variation among genera in estimated modeled effective rooting depth as differences observed among these sample mean estimates were not statistically significant (*P* = 0.12). Moreover, there was not a systematic linear relationship between effective rooting depth and soil depth (*P* = 0.30). When modeled effective rooting depth was restricted to the depth of soil water that was sampled (30 cm), the grand mean effective rooting depth was just 6 ± 1 cm. This shallow effective rooting depth estimate could highlight the importance of shallow soil water from warm-weather precipitation to tree water sources in this catchment. Taken together, these two effective rooting depth estimates (32 and 6 cm) could represent the influence of multiple depths of tree water sources.

### Soil moisture depletion

**Figure 7.**
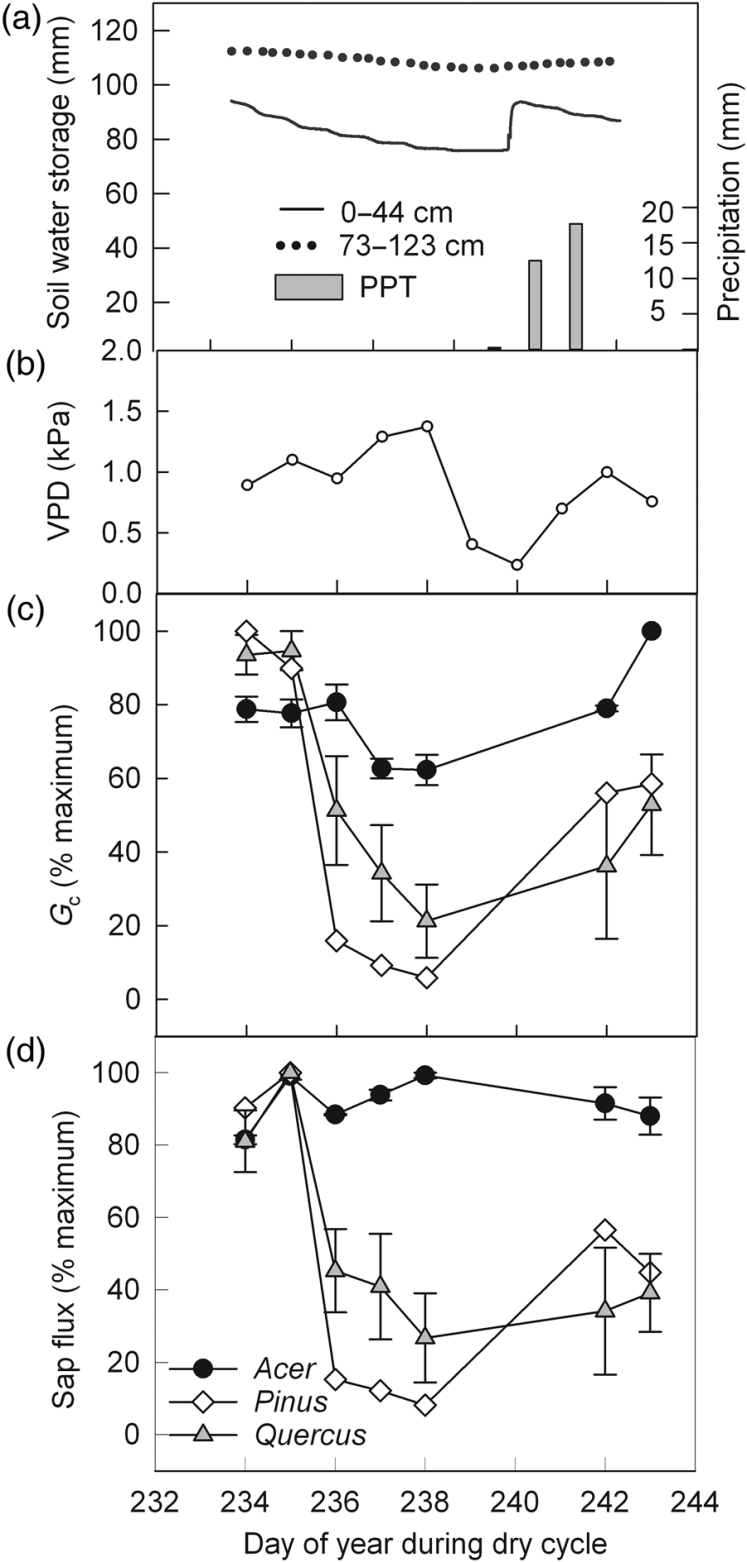
(a) Soil moisture depletion and precipitation during a soil drying and rewetting cycle for one hillslope site in August 2009. Total soil water from 0 to 44 cm (closed circles) and 73–123 cm (open circles) is shown. Values were weighted by midpoint between sensor depths. (b) Daytime (7 am to 7 pm) VPD in kPa for the dry week and recovery, with values <0.1 kPa omitted. (c) Crown conductance (*G*_c_,% maximum) based on sap flux and VPD for *Acer*, *Quercus* and *Pinus* individuals at the south ridge site during the same soil drying and rewetting cycle. (d) Sap flux (% maximum) for individuals at the south ridge site. For (c) and (d), values for individuals of each genus, *Quercus* (*n* = 3) and *Acer* (*n* = 2) are shown with bars representing standard errors of the mean (*Pinus*, *n* = 1). Average values for crown conductance and sap flux were normalized by the percentage of maximum over the dry cycle and recovery. Day of year 239, 240 and 241 were omitted due to precipitation events.

Isotopic-based estimates of mean depth of soil water extraction should correspond to patterns of soil moisture depletion. If trees were using significant amounts of deep water, we expected to observe sizable depletions in soil moisture at ≥1 m. The average amount of depletion of soil water storage over a dry week in July 2009 for three hillslope sites was 10.5 ± 1.6 mm for depths shallower than 40 cm and 3.8 ± 0.5 mm for depths >40 cm (Figure 7a). The rate of soil moisture depletion slowed by the end of the dry week before rewetting occurred, which provided evidence of decreasing soil moisture availability in the shallow layers. Similarly, average volumetric water content (m^3^ m^−3^) at the same locations declined significantly over the dry cycle in 2009 (*P* < 0.01) at all sensor depths measured. Soil moisture at depths shallower than 40 cm declined the greatest (10.2, 14.3 and 7.1% depletion for 0–10, 10–20 and 20–40 cm depths, respectively) with minor depletions observed in the deeper soil (3.9, 2.0 and 1.3% decline for 60–80, 80–100 cm and over 100 cm, respectively) (see Figure S3 available as Supplementary Data at *Tree Physiology* Online).

### Sap flux

The relationship between sap flux and VPD varied among individuals from different genera (Figure 7d). The sap flux of *Acer* individuals broadly followed trends in VPD—as VPD increased during the dry part of the week, *Acer* sap flux also increased. In contrast, sap flux in *Pinus* and *Quercus* individuals declined sharply during the dry period, despite an increase of ∼34% in VPD over the same period (Figure 7b). Overall, sap flux declined by ∼62% in *Quercus* and 89% in *Pinus* and increased by 7% in *Acer* from the start to end of the dry cycle (DOY 234–235 versus DOY 237–238) (Figure 7d). This uncoupling between sap flux and VPD, in the context of declining soil moisture (Figure 7a), suggested that low soil moisture content may have contributed substantially to the decline of sap flux, at least for *Quercus* and *Pinus* individuals. Mean daily crown conductance, which provided an index of sap flux that was normalized by VPD, also declined by 59% on average over the dry period with modest declines in *Acer* and sharp drops in *Pinus* and *Quercus*.

Declines in sap flux were also noted in 2010, but with different trends by genera. Over four dry cycles of at least 5 days in duration, six individuals from a ridge top site averaged sap flux declines of 17%. The *Quercus* were less responsive to soil drying with a net gain of 5% in mean daily sap flux, whereas the *Acer* individuals measured declined by 38% on average. Crown conductance declined modestly in *Acer* during dry days and increased after rain, but dropped sharply in *Pinus* and *Quercus*.

## Discussion

Effective rooting depth has not been well studied in humid temperate forests. Although probabilities of deep rooting are low for cool temperate climates (Schenk and Jackson 2005), the frequent observation of roots at depths greater than a meter in forests of the northeastern USA (e.g., Dawson 1993) including at Shale Hills through trenches (Brantley et al. 2014, personal observation) and deep soil cores (T.S. Adams, unpublished data) suggested that this paradigm deserved closer examination. In this study, we did not see strong evidence that trees were regularly accessing deep soil water (>1 m) or that groundwater contributed appreciably to the water balance of trees in the study. Instead we found evidence in support of predominantly shallow root distributions and the majority of water uptake at less than ∼60 cm depth, with some differences in xylem water isotopic composition related to tree genus, size and, to a lesser degree, soil depth.

Root length density measurements showed that the majority of roots were distributed in the top 40 cm regardless of slope position or soil depth. Additionally, water sources based on natural abundance of oxygen and hydrogen isotopes were estimated to be on average ∼32 cm deep with a large amount of variation (95% confidence interval, 15–61 cm). When 30 cm was used as an end member for the mixing model, water uptake was estimated at just 6 cm deep. One possible explanation for the lack of evidence of deeper soil water extraction is that the trees had sufficient water supplies in the shallow soil layers to meet their needs, negating the need to access deeper water. However, corroborative evidence from soil moisture depletion at depth and sap flux patterns also suggested a predominance of shallow water use. We observed greater soil water depletion in the shallow soil layers (≤40 cm deep) than the deeper soil layers (>73 cm deep), with the rate of depletion slowing as the upper soil layers dried (Figure 7). Sap flux measurements during periods without rainfall suggested a tight coupling of transpiration with precipitation.

Species and genus effects on effective rooting depth have been well studied in seasonally dry and arid climates (Moroni et al. 2003, Poot and Lambers 2003, Viola et al. 2008), but less so in humid temperate climates. Species-level differences have been noted for both vertical root access and lateral root elongation and proliferation that could lead to greater water access through foraging in rock channels and fissures (Poot and Lambers 2003). We found evidence of deeper rooting among *Quercus* and *Carya* species than in *A. saccharum* through relative isotopic differences in δ^18^O and δ^2^H compositions. We were able to verify these differences statistically in 2011 using pairs of *Q. prinus* and *A. saccharum* individuals sampled on the same days and in close proximity to one another. *Quercus* species have been found to be deep rooted in a variety of ecosystems (Biswell 1935, Abrams 1990, Stone and Kalisz 1991, Phillips and Ehleringer 1995, Canadell et al. 1996, Jackson et al. 1999, McElrone et al. 2007), and *Carya* has also been shown to have a deep taproot (Burns and Honkala 1990). The *Quercus* and *Carya* spp. in this study showed evidence, through xylem water isotopic compositions, of accessing different water sources than *A. saccharum* by tapping deeper water. This result was also confounded by tree size effects, with *Acer* tending to be smaller than trees in other genera.

The overall relationship between δ^18^O and δ^2^H with DBH and height was consistent with other work showing that tree size may be an important factor related to depth of water uptake. Typically, larger trees have been shown to use deeper water (Phillips and Ehleringer 1995, Dawson 1996, Goldsmith et al. 2012), although the opposite relationship has also been observed (Meinzer et al. 1999). The wider range in sizes of *A. saccharum* individuals sampled may have enhanced the relationship in this genus. These results highlight the importance of species composition within a forest, which could affect the relationship between tree size and depth of water acquisition.

Topography had a less clear effect on effective rooting depth. Tree water δ^18^O and δ^2^H did vary based on soil depth, but not by slope position. It is important to note that this variation with soil depth likely did not reflect differences in effective rooting depth, but rather differences in δ^18^O and δ^2^H composition with contrasting soil conditions. Further, it is quite possible that the soil depth at the location of the trees sampled was not representative of all of the roots for a given tree, given the lateral spread of roots and the uneven topography (presence of swales, for example). Given the variation of soils within the catchment (Baldwin 2011), soil-based factors like texture or pore size likely influenced the isotopic variation in soil water that was observed, relative to catchment location. Other key soil properties may have independently promoted shallow rooting on different slope positions. For example, the fragipan-like redoximorphic feature from seasonal flooding in some regions of the valley floor may have inhibited deeper rooting or caused high fine root mortality. In addition, higher nutrient availability near the surface could have favored shallow root development, particularly on the rocky soils on hillslopes and ridge areas. Decreasing nutrient and oxygen availability (e.g., Goldsmith et al. 2012) with depth have both been shown to favor shallow rooting in many ecosystems, in addition to greater competitive ability of shallow roots, and lower construction and maintenance costs (Schenk 2008). Once the differences in isotopic compositions of bulk soil water between slope positions were accounted for in a linear mixing model in this study, the between-slope position differences in effective rooting depth were eliminated.

Further evidence of limited direct influence of topography on effective rooting depth was found in our investigation of root length across slope positions. Despite our expectation that root length would be greatest in the deep soils at the valley floor where trees were the largest, we were not able to detect differences in root length density among trees located at different slope positions.

The tree xylem water δ^18^O and δ^2^H composition showed evidence of an evaporative signal when compared with the amount-weighted LMWL. In this dual isotope space, this composition was consistent with a shallow, bulk soil water signal. The evaporative signal observed in tree water is consistent with a small body of work from other ecosystems, where tree water also showed evidence of originating from an isotopically separate pool from groundwater or stream water (Brooks et al. 2010, Goldsmith et al. 2012). This water may be held at tensions between that of water sampled by suction lysimeters (<0.06 MPa) and by cryogenic vacuum distillation (<15 MPa) (McDonnell 2014), and may be used by trees late in the growing season after mobile soil water is exhausted. This phenomenon is still an area for further research (McDonnell 2014), but it may indicate that trees are using water stored in the soil profile from an earlier season or year. The large amount of variation we observed in tree δ^2^H and δ^18^O compositions itself suggests that trees were using water from a highly variable water source, which points to shallow soil water rather than deep soil water or groundwater as the main source of water supporting transpiration.

It should be noted that the rooting depths we provide here represent rough estimates. Given the complexity of a root system, roots in different locations could be using water from different sources and held under different tensions. As a result, the depths presented here represent an average depth of water use. To further emphasize this point, we have reported estimates for depth of water use with two alternative models—one with 120 cm and a second with 30 cm as the maximum depth of water uptake. The results of both models point to quite shallow water use throughout the catchment, with considerably shallower water use implied when 30 cm is used as a mixing model end member. Despite the overall evidence pointing to quite shallow water use throughout the growing season in this humid, temperate forest, a small amount of deep water use likely occurs and may have important consequences for tree physiology and survival. In addition, it is possible that over longer time frames without precipitation, deeper water use could increase, particularly as severe drought events are expected to increase in frequency over the coming years (Prudhomme et al. 2014). The trend we observed in 2010, with *Quercus* showing a net increase in sap flux during dry periods, suggests that deeper water use could occur in this ecosystem during very dry years, although this may not be typical. The exploitation of deeper soil water as the season progresses has been observed in a number of systems including coniferous forests of the Pacific Northwest (Warren et al. 2005, Meinzer et al. 2007), mixed-oak forest in France (Bréda et al. 1995), Australian woodlands (Mensforth et al. 1994, Dawson and Pate 1996, Burgess et al. 2000) and a tropical forest in Panama (Meinzer et al. 1999). These systems also had strong seasonal differences in soil water content, with a marked dry season during which deep root function was observed. The sites shared deep soils (2 m or deeper), often with a high sand content. We would expect that systems with strong seasonal plasticity in depth of water uptake would be more resilient to drought, and quite possibly more productive than those with uniformly shallow water uptake, given their ability to maintain or even increase transpiration when tapping deeper water sources (Meinzer et al. 1999). The current precipitation regime in central Pennsylvania likely dampens this type of plasticity in depth of water uptake, particularly in a growing season with typical, or greater than average precipitation.

This lack of deep root function could have implications for other humid temperate forested areas and may be important for hydrologic process models. This study may also indicate that processes like hydraulic redistribution are unlikely to be a major contributor to similar systems with shallow soils, which could be important for modeling. Trees with primarily shallow roots are unable to reach deep moist soil or to build dimorphic root systems that are typically considered necessary for hydraulic redistribution (Pate et al. 1995, Dawson and Pate 1996, Hultine et al. 2003, Scholz et al. 2008).

Much work is still needed in the area of ecohydrology of humid temperate forests including gaining a deeper understanding of the spatial patterns and drivers of soil and tree water isotopic signatures. Another important area for research is characterizing the distribution and function of roots at the root–rock interface and the isotopic compositions of water held within rock fractures and rocks themselves.

In conclusion, for trees located on both shallow and deeper soils, we found little evidence that roots located within or below fractured bedrock were consistently major contributors to transpiration. Although there was some variation among genera and among locations varying by soil depth, the depth of tree water uptake was generally quite shallow. Further, xylem water was isotopically variable, and unlikely to originate from an isotopically stable, deep water source.

## Data accessibility

Data used in the manuscript and original datasets for water sources and environmental data are available at http://criticalzone.org/shale-hills/, including tree water isotopes (Gaines and Eissenstat 2011), root length density (Eissenstat 2013*a*), precipitation amount (Duffy 2013), air temperature (Davis and Shi 2013), soil moisture (Lin 2012), sap flux (Eissenstat 2013*b*), groundwater isotopes (Duffy and Thomas 2011*a*), precipitation isotopes (Duffy and Thomas 2011*b*), soil water isotopes (Duffy and Thomas 2011*c*) and vegetation survey (Eissenstat 2012).

## Supplementary data

Supplementary data for this article are available at *Tree Physiology* Online.

## Conflict of interest

None declared.

## Funding

Financial and logistical support and data were provided by National Science Foundation Grant EAR 07-25019 (C. Duffy), and EAR 12-39285 and EAR 13-31726 (S. Brantley) for the Susquehanna Shale Hills Critical Zone Observatory. Financial support during the creation of this manuscript was provided by National Science Foundation Grant CarbonEARTH (09-47962) to K.P.G. Funding to pay the Open Access publication charges for this article was provided by The National Science Foundation.

## Supplementary Material

Supplementary Data
