# Supplementary material for: Reliance on shallow soil water in a mixed-hardwood forest in central Pennsylvania
Source: Tree Physiol. 2015 Nov 6;36(4):444–58. doi: 10.1093/treephys/tpv113 (PMC4835221; doi:10.1093/treephys/tpv113)
Supplement: Supplementary Data [file supp_36_4_444__index.html]

Reliance on shallow soil water in a mixed-hardwood forest in central Pennsylvania — Supplementary Data 

# Reliance on shallow soil water in a mixed-hardwood forest in central Pennsylvania

## Supplementary Data

Supplementary Data

- Supplementary Data - Docx file
