# Supplementary material for: Reliance on shallow soil water in a mixed-hardwood forest in central Pennsylvania
Source: Tree Physiol. 2015 Nov 6;36(4):444–58. doi: 10.1093/treephys/tpv113 (PMC4835221; doi:10.1093/treephys/tpv113)
Supplement: Supplementary Data [file supp_tpv113_tpv113supp.docx]

Supplemental Figures


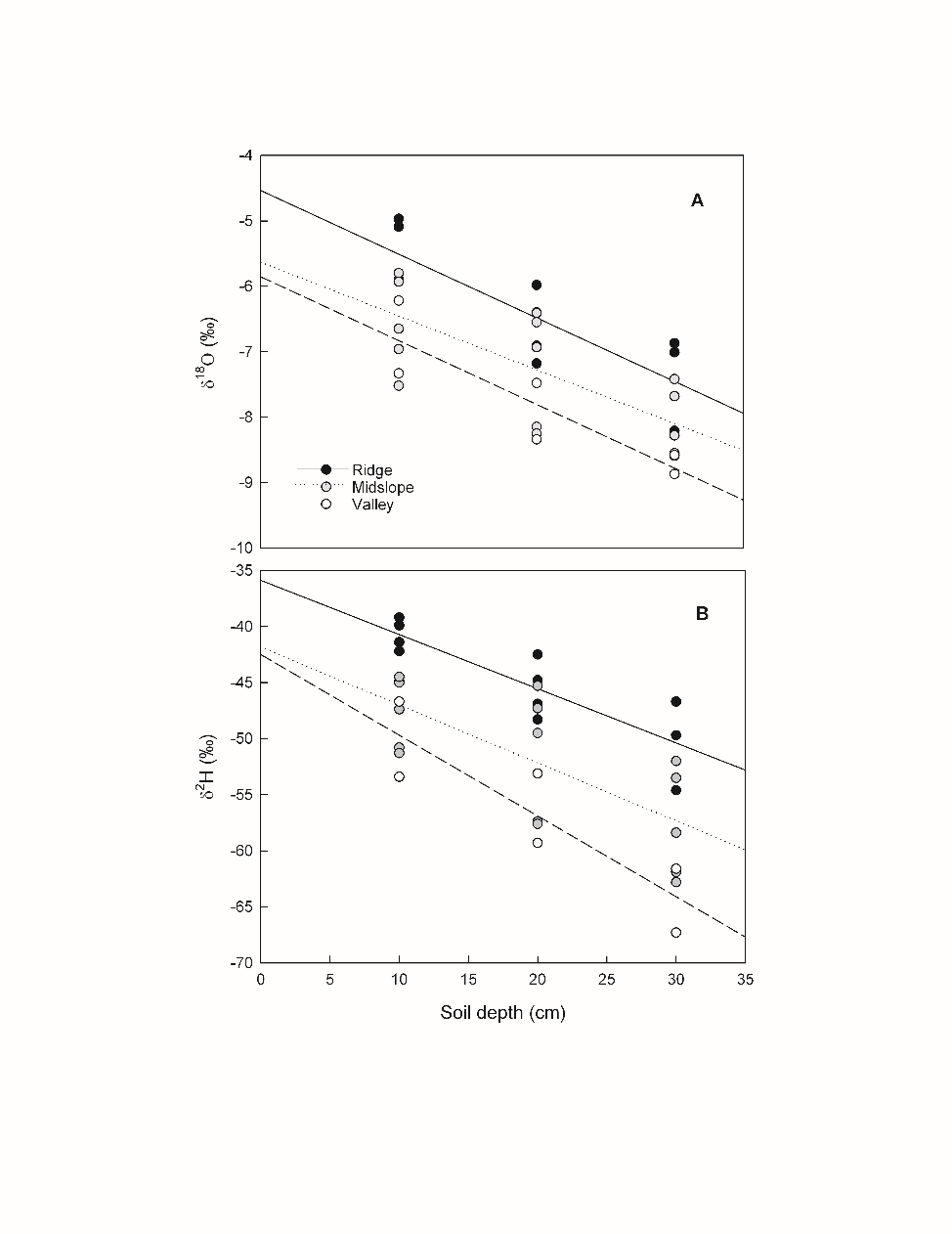


Figure S1. – Bulk soil water δ^18^O (A) and δ^2^H (B) versus depth for 12 sites sampled in July 2012. Linear regressions for each slope position were used to estimate the δ^18^O and δ^2^H compositions at 0 cm depth to include as end members for linear mixing models to calculate effective rooting depth. (A) ridge, y = ‑0.0973x - 4.5351, *R²* = 0.70; midslope, y = ‑0.0821x - 5.6318, *R²* = 0.53; valley, y = ‑0.0975x - 5.8533, *R²* = 0.78. (B) ridge, y = ‑0.4836x - 35.878, *R²* = 0.74; midslope, y = ‑0.517x - 41.833, *R²* = 0.51; valley, y = ‑0.72x - 42.5, *R²* = 0.78.


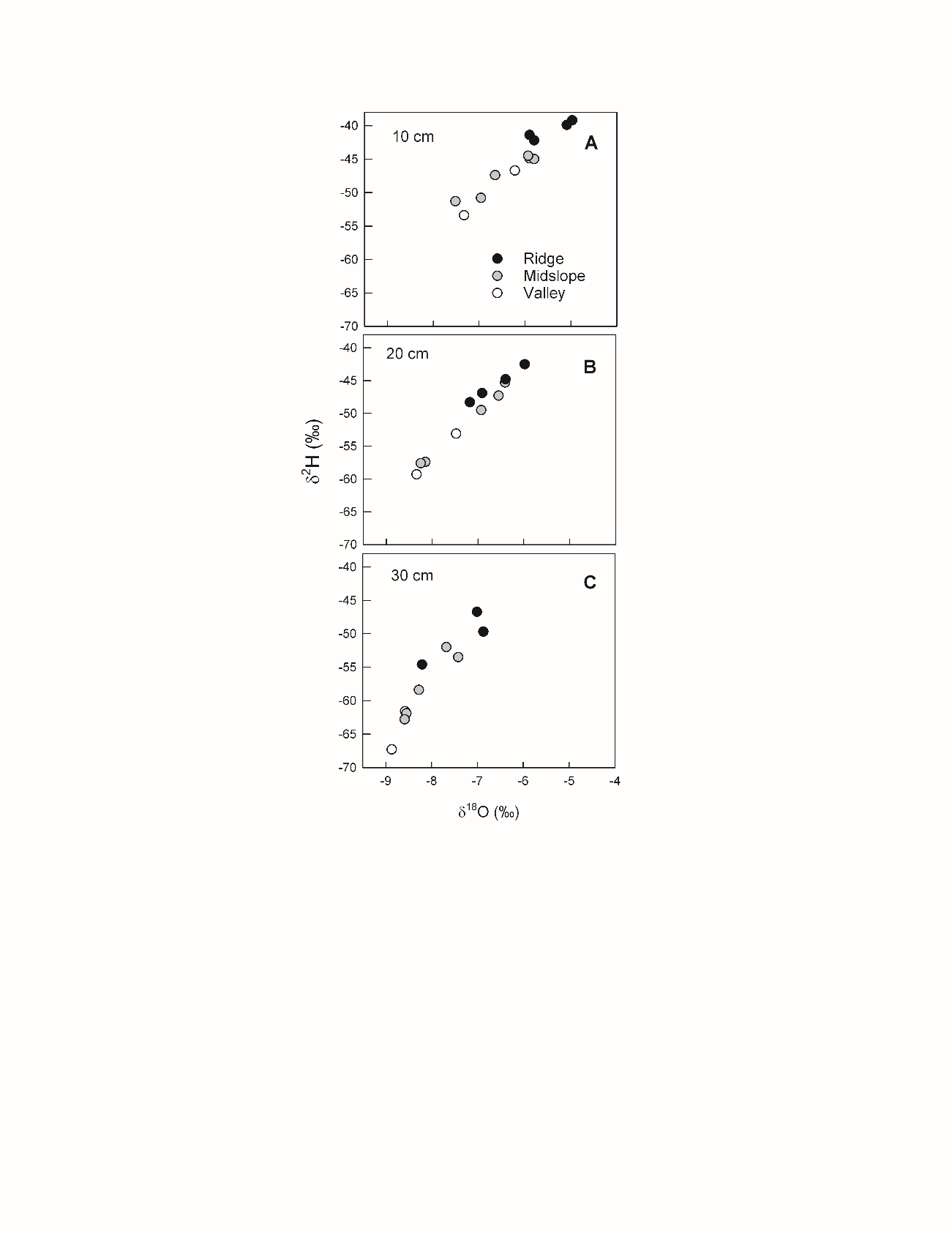


Figure S2. – Bulk soil water scatter plot of δ^2^H and δ^18^O by depths of 0-10 cm (A), 10-20 cm (B) and 20-30 cm (C) for trees sampled on ridge (*n* = 4 ), midslope (*n* = 6) and valley sites (*n* = 2). Samples were from 12 sites sampled in July 2012.


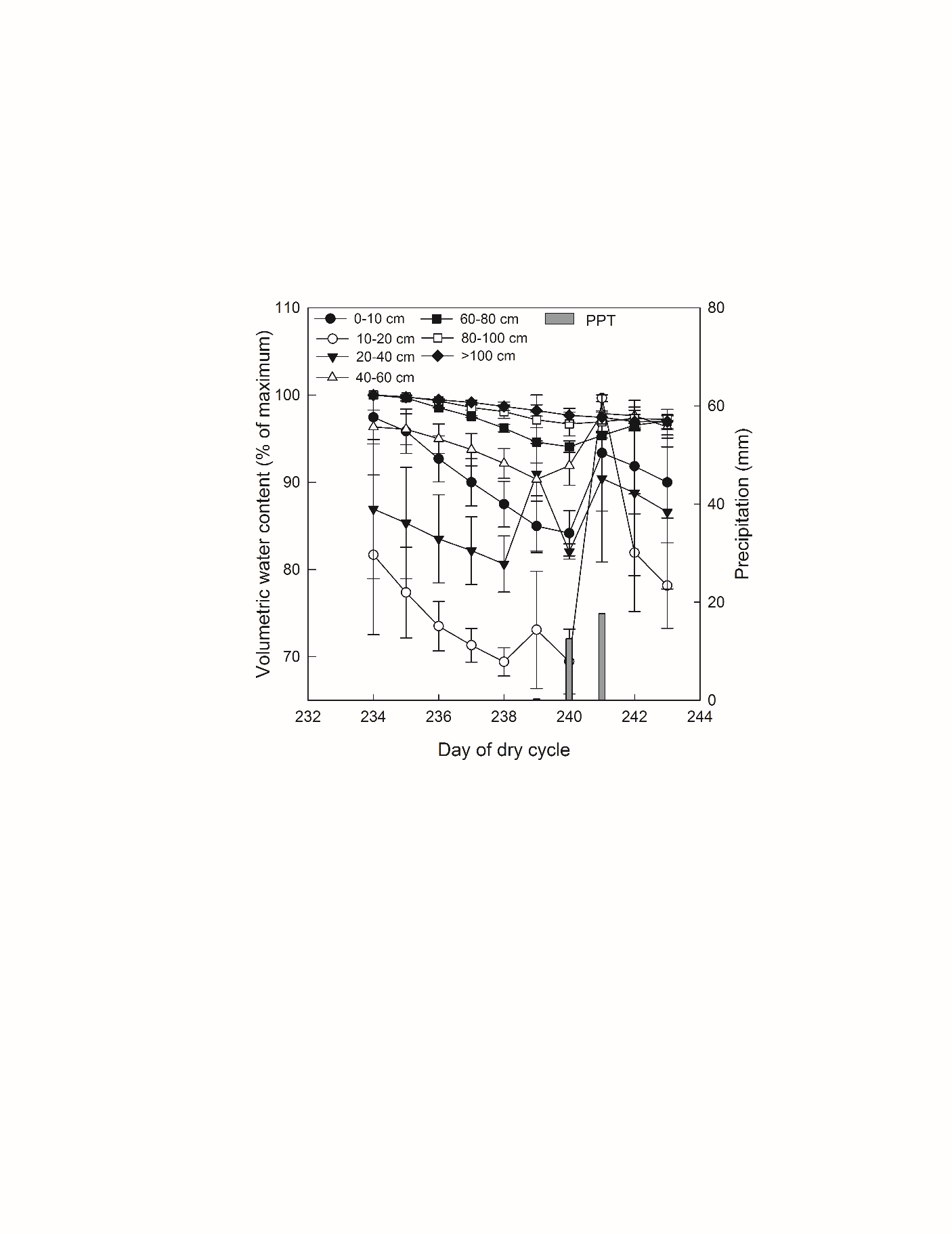


Figure S3. – Hill slope soil moisture content during a soil drying and rewetting cycle in August 2009. Average volumetric water content values between three soil moisture sensors and depth categories are shown, with maximum daily values normalized by the percent of maximum soil moisture for the one-week cycle. Dry days were days of year 233 to 238 with precipitation beginning again on day of year 239. For the period without rain, there was a significant negative linear relationship between maximum daily volumetric water content and day of dry period (*P*<0.01).
